# Acute Flaccid Myelitis Caused by West Nile Virus: A Case Report and Neuroimaging Correlate

**DOI:** 10.7759/cureus.70107

**Published:** 2024-09-24

**Authors:** Aaron Creswell, Cortney M Connor, Raymond Ko, Sally Tu, Shahnawaz Karim, Forshing Lui

**Affiliations:** 1 Neurology, California Northstate University College of Medicine, Elk Grove, USA; 2 Neurology, Kaiser Permanente, Sacramento, USA; 3 Clinical Sciences, California Northstate University College of Medicine, Elk Grove, USA

**Keywords:** acute flaccid paralysis (afp), ascending flaccid paralysis, asymmetric paralysis, neuroinvasive west nile virus, west nile, west nile virus encephalopathy

## Abstract

West Nile virus (WNV) is the most common mosquito-borne illness in the United States. Most cases remain asymptomatic or may be associated with a mild febrile illness; however, it can invade the central nervous system and cause meningoencephalitis, or rarely, acute flaccid paralysis (AFP). Here, we describe a case of WNV-associated paralysis in a previously healthy male presenting with asymmetric weakness and absent deep tendon reflexes. Magnetic resonance imaging (MRI) of the spine displayed a hyperintensity lesion restricted to the central gray matter, preferentially affecting the ventral horns, which is reflected by his clinical features. This case contributes to mounting evidence that WNV can cause selective injury to the ventral gray matter of the spinal cord and demonstrates that WNV should be considered a unique causative agent in patients presenting with AFP.

## Introduction

West Nile virus (WNV) is a vector-borne flavivirus transmitted by the common urban mosquito, Culex pipiens, and the floodwater mosquito, Aedes vexans, and is widely endemic to North America, Europe, Africa, the Middle East, and West Asia. Although WNV is the leading cause of mosquito-borne illness in the United States, 70-80% of cases are asymptomatic, with most symptomatic individuals presenting with flu-like symptoms, gastrointestinal distress, lymphadenopathy, or a petechial or maculopapular rash [1]. In rare instances thought to be associated with hematological dissemination and inflammatory impairments in the blood-brain barrier [2-5], the virus can invade the central nervous system (CNS) and infect both neurons and glial cells. This may produce symptoms consistent with encephalitis and/or meningitis, including nuchal rigidity, altered mental status, cranial nerve dysfunction, seizure, or even coma [6]. A tropism for cells in the basal ganglia has been well described, which manifests as muscle rigidity, tremor, and other motor disturbances [7]. More recently, WNV infection has been associated with injury to anterior horn cells within the spinal cord, which causes acute flaccid paralysis (AFP), referred to as WNV poliomyelitis-like syndrome. This phenomenon strongly resembles an infection with poliovirus, with most cases exhibiting asymmetric weakness, impaired reflexes, and intact sensation following a typical prodrome of WNV infection and other signs of neuroinvasion [8]. This article was previously presented as a meeting abstract at the 2024 American Academy of Neurology Annual Meeting on April 17, 2024.

## Case presentation

All information in this case report is de-identified. Informed consent has been obtained from the patient for publication purposes. The present report describes a previously healthy 42-year-old male who presented to the emergency room with acute-onset back pain, truncal weakness, and difficulty walking following a six-day history of fever recorded between 99 and 101F (37.2-38.3C). He denied cough or sputum production, as well as any skin rash or gastrointestinal or genitourinary disturbances. Recent travel and sick contacts were noticeably absent from his history; however, the patient stated he went hiking in the local foothills 10 days ago but did not recall receiving any bug bites. Physical examination revealed an alert and oriented male with a temperature of 102.8F, blood pressure of 130/78 mmHg, pulse of 102/min regular rhythm, and oxygen saturation of 98% on room air. Cardiac, pulmonary, and abdominal exams were all normal. Head and neck exams revealed no nuchal rigidity or cervical lymphadenopathy. Examination of the back showed no tenderness and a normal range of motion. Neurological examination demonstrated a negative Brudzinski sign and a Kernig sign. There was no evidence of neuropsychiatric abnormalities or other focal neurological deficits such as aphasia or dysarthria, and all cranial nerves were intact. The patient displayed grade ⅗ weakness in the left hip flexors and knee extensors with normal strength elsewhere. Sensation was normal to light touch and pinprick; however, the left knee jerk reflex was absent with all other reflexes at 2+, and the plantar response was flexor bilaterally. He dragged his left leg when he walked. Coordination was normal with finger-to-nose and heel-to-shin testing. Romberg's test was negative.

**Figure 1:**
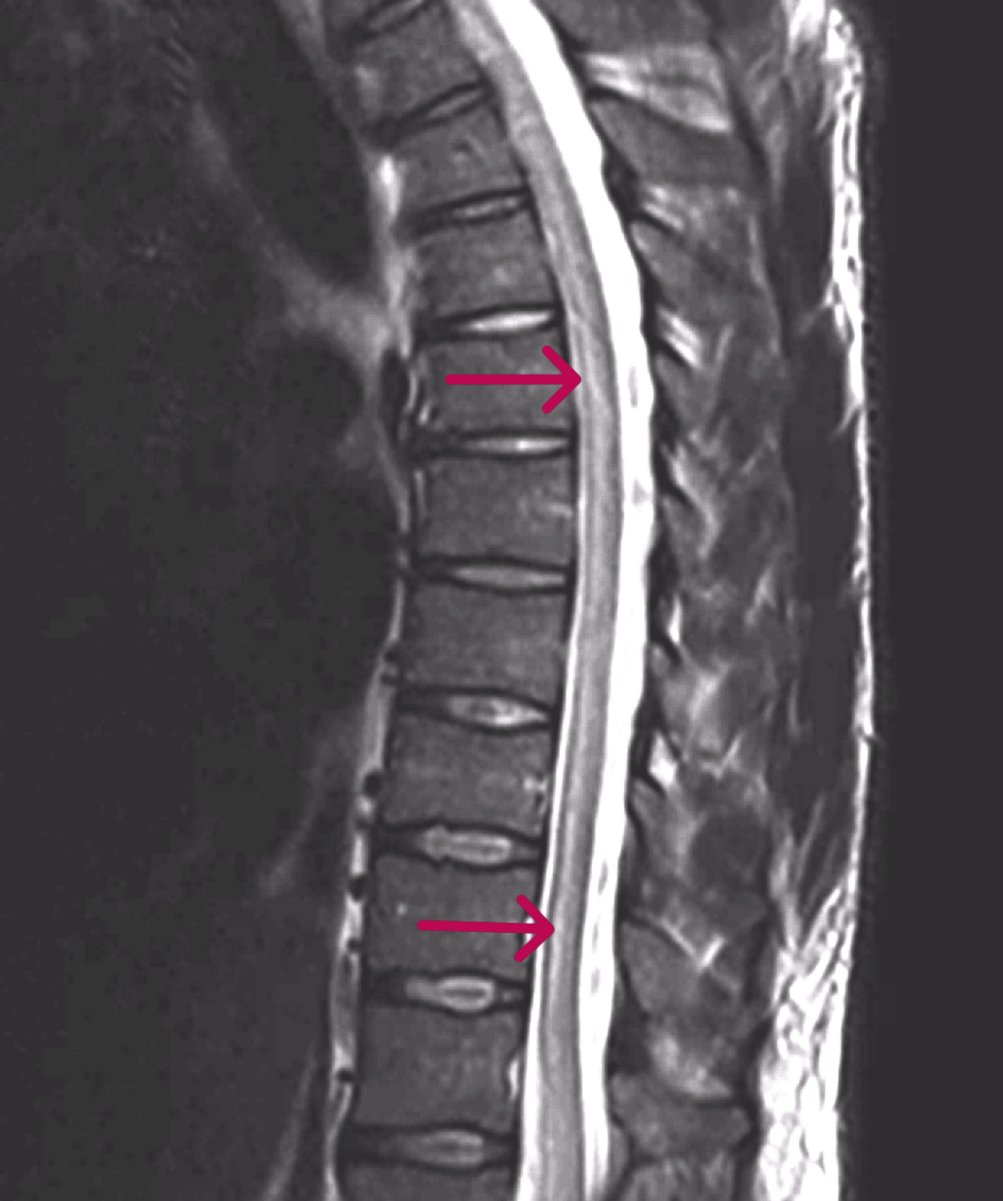
T2 weighted MRI of sagittal spine displaying gray matter lesion from T7 extending to conus medullaris. Arrows indicate involvement of the central gray matter

**Figure 2:**
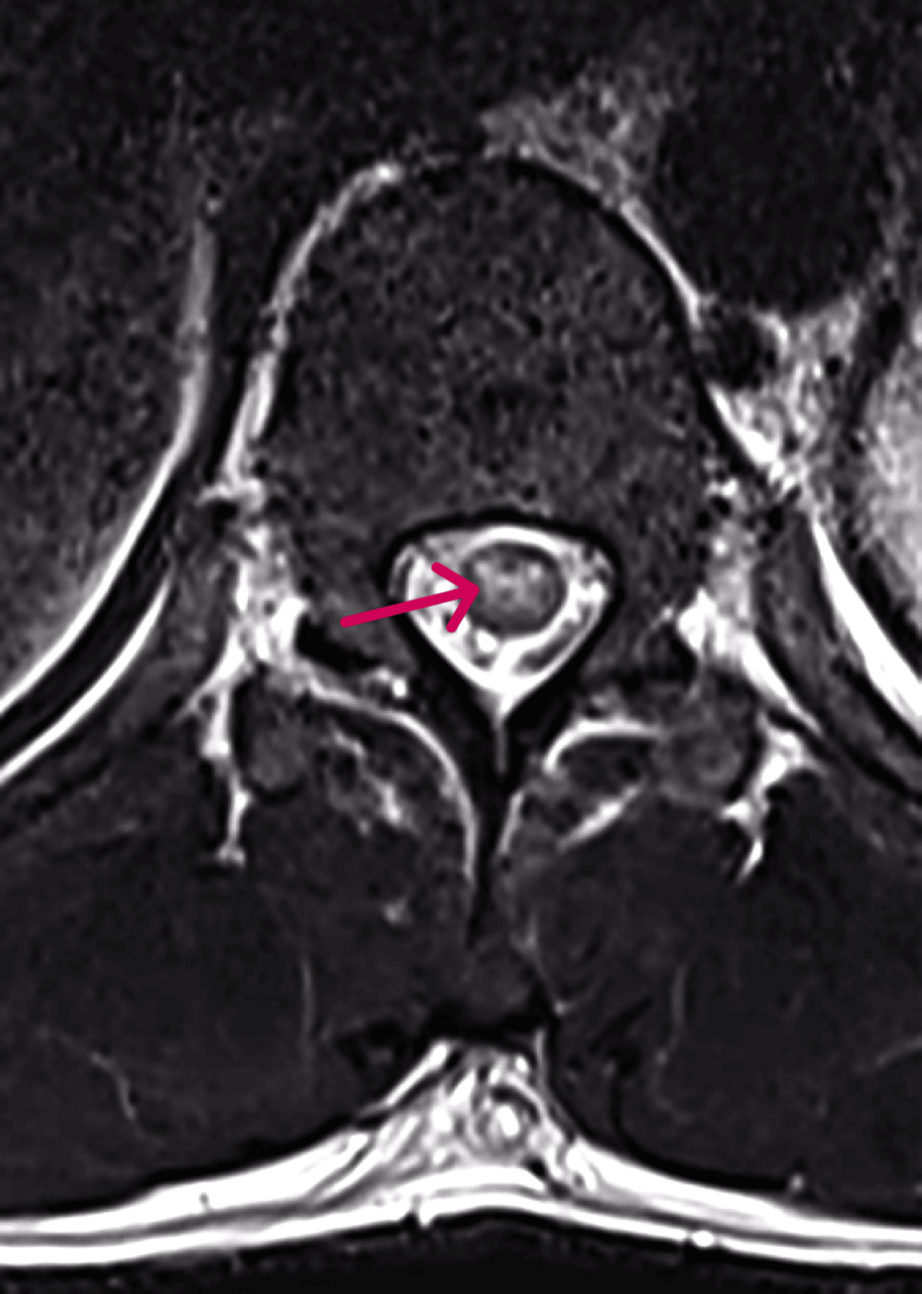
T2 weighted MRI of axial spine indicating pathology restricted to central gray matter within the spinal cord

Laboratory studies showed normal complete blood counts with normal differential white cell counts. His alanine aminotransferase (ALT), aspartate aminotransferase (AST), serum albumin, calcium, and alkaline phosphatase were all within normal limits. Corticospinal fluid (CSF) was obtained and contained no red cells, 265 white cells per ml, 47% polymorphs, and 44% lymphocytes. The CSF glucose was normal, and protein was elevated to 106 mg/dL (Table 1). A subsequent CSF result was found to be positive for WNV IgG (Table 1), and the serum contained WNV IgG at 4.3 mg/dL and a WNV IgM of 6.33 mg/dL, both of which are positive (Table 2). Magnetic resonance imaging (MRI) of the brain was normal. A spine MRI showed a linear hyperintensity lesion beginning at the level of T7 and extending to the conus medullaris, restricted to the central gray matter of the spinal cord (Figure 1). Strikingly, an axial MRI displayed selective involvement of the ventral gray matter, sparing the long tracts (Figure 2). On follow-up after several weeks, the patient continues to experience debilitating weakness and backaches.

**Table 1:** CSF analysis CSF: corticospinal fluid; WNV: West Nile virus

Parameter	Patient value	Reference range
Leukocyte count	265 x10^3^/mL	0-4 x10^3^/mL
Erythrocyte count	0 /mm^3^	<1 /mm^3^
Protein	106 mg/dL	<45 mg/dL
Glucose	52 mg/dL	40-70 mg/dL
WNV IgG	Positive	Negative

**Table 2:** Serum serology WNV: West Nile virus

Antibody	Patient value	Reference range
WNV IgG	4.3 mg/dL	<1.3 mg/dL
WNV IgM	6.33 mg/dL	<0.9 mg/dL

## Discussion

Since its introduction to North America in 1999, WNV has become the single most common cause of viral encephalitis in the US [9]. While most cases of WNV infection remain subclinical, the Center for Disease Control and Prevention reports almost 2000 cases of neuroinvasive infections in 2023 alone [10], which can cause an array of symptoms, including altered mental status, slurred speech, and muscle weakness. Brain imaging studies of these patients have demonstrated a variety of MRI abnormalities throughout the cortex and brainstem, with non-specific findings affecting the basal ganglia, thalamus, cerebellum, or white matter, often resembling infarction [11]. The occurrence of AFP associated with WNV has gained increasing attention following more recent outbreaks, due in part to its strong likeness to the presentation of poliomyelitis [12]. Spinal MRI of WNV-infected patients suffering from AFP has also revealed some variation and non-specificity; however, a predilection has been described for lower motor neurons within the gray matter of the ventral spinal cord, conus medullaris, and cauda equina [13-15].

Our patient presented with a clinical picture most compatible with acute inflammatory meningomyelitis, with a six-day history of fever and back pain followed by rapidly progressive flaccid paralysis. The most striking feature of our patient is the axial spinal MRI showing selective involvement of the central gray matter, primarily affecting the ventral horns with some asymmetry. These findings are reflected in his clinical presentation of left-sided AFP.

A variety of bacteria, fungi, and parasites have been associated with CNS infiltration and subsequent paralysis, as well as viruses such as polio, enteroviruses, and Coxsackie B [16], which would produce a CSF profile consistent with that of our patient. CSF serology remains the definitive method to determine the causative agent. In addition, Guillain-Barre syndrome (GBS) is the most common cause of AFP worldwide and is characterized by post-viral inflammation that induces demyelination of peripheral nerves, causing symmetric ascending paralysis, impaired sensation, decreased deep tendon reflexes (DTRs), and some cases autonomic nerve dysfunction [17]. The asymmetry and absence of sensory deficits observed in our patient are key clinical elements distinguishing from this syndrome; however, GBS was a preliminary diagnosis in many previous reports of WNV myelitis [18-21], and in fact, some cases of neuroinvasive WNV have resulted in demyelinating processes consistent with GBS [22-24]. Therefore, the diagnosis of WNV-related AFP requires careful differentiation between inflammatory peripheral demyelination and CNS injury, using a combination of clinical features, electromyographic tests, serology studies, and imaging. The present study contributes to mounting evidence that active WNV infection can cause injury to cells within the anterior horn of the spinal cord comparable to poliomyelitis, resulting in asymmetric weakness and absent DTRs. Given this distinct pathophysiology, typical treatments for GBS, including intravenous immunoglobulin or plasmapheresis, which are occasionally administered empirically prior to diagnostic certainty, are inappropriate and in fact, have been proven ineffective [18-20]. Unfortunately, though most individuals with non-neuroinvasive WNV or even WNV meningitis recover completely from their illness, those with WNV encephalitis or myelitis most often suffer from persistent neurologic deficits and are at greater risk of fatality [25]. Treatment options are currently limited to supportive therapy.

## Conclusions

This case comprises one of exceedingly few reports of WNV complicated by AFP and confirms that WNV infection can lead to irreversible injury to lower motor neurons in the ventral horns of the spinal cord and conus medullaris. In conclusion, neuroinvasive WNV should be considered as a cause of isolated AFP, especially in the late summer months.
